# Immunogenicity profile after COVID-19 vaccination in patients with onco-hematological diseases

**DOI:** 10.31744/einstein_journal/2023AO0089

**Published:** 2023-03-07

**Authors:** João Bosco de Almeida, Inara Lúcia Arce, Vera Lúcia de Piratininga Figueiredo, Perla Vicari

**Affiliations:** 1 Hospital do Servidor Público Estadual de São Paulo São Paulo SP Brazil Hospital do Servidor Público Estadual de São Paulo , São Paulo , SP , Brazil .

**Keywords:** COVID-19, Coronavirus infections, SARS-CoV-2, Immunogenicity, Vaccines, Myelodysplastic-myeloproliferative diseases, Seroconversion, Lymphoproliferative disorders, Multiple myeloma

## Abstract

**Objective:**

To evaluate the influence of onco-hematological pathologies on seroconversion to COVID-19 vaccines, in addition to the effects of chemotherapy treatment on this response.

**Methods:**

The present study evaluated the immunogenic response of 76 patients with onco-hematological diseases to multiple vaccine platforms compared to 25 control individuals.

**Results:**

Our results showed positive response rates of 74.02% in patients with onco-hematological diseases and 100% in controls. When analyzed according to etiological group, patients with lymphoproliferative disorders achieved a positive vaccine response rate of 58.7%, whereas those with myeloproliferative diseases achieved a 100% response rate. We also observed that patients previously exposed to COVID-19 presented a 75% increase in their antibody values after vaccination, and these values were 37% higher than those of patients who did not have such exposure. We found that patients who underwent B-lymphocyte-depleting therapy in the last 2 years before vaccination had a worse response rate of 18.75%.

**Conclusion:**

Despite the immunosuppression of patients with onco-hematological diseases, caused by the biology of their diseases and treatment, benefit and safety in vaccinating these patients are observed, in view of the important recall immune response and incidence of adverse effects similar to those of the healthy population.

**Figure:**
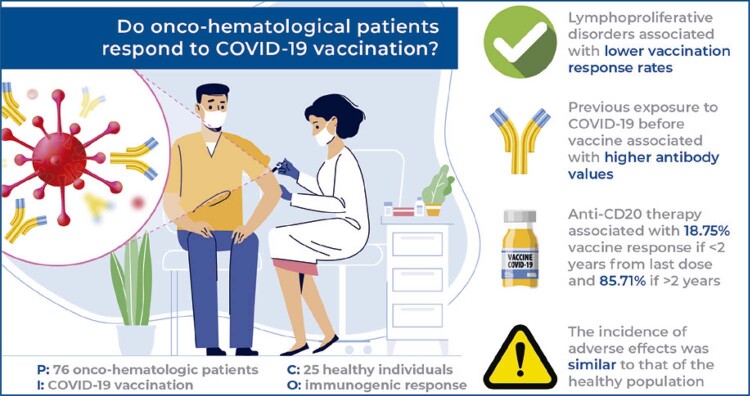

## INTRODUCTION

The coronavirus disease 2019 (COVID-19) pandemic has cost countless lives and resources. Its symptoms are heterogeneous, ranging from asymptomatic individuals to acute respiratory failure requiring ventilatory support. ^( 1 , 2 )^ Today, there are some drugs authorized in Brazil for use during COVID-19, such as the monoclonal antibodies regdanvimab, casirivimabe, imdevimabe, banlanivimabe, and etesevimabe, and the antiviral drug remdesivir. Additionally, there are studies investigating the role of other antiretroviral medications in the treatment of COVID-19. The main drugs studied in this regard are molnupiravir, paxlovid, and fluvoxamine. A recent meta-analysis demonstrated a 56% reduction in mortality and an 80% reduction in hospitalizations with these drugs when compared with placebo. ^( 3 )^ There are also some protocols that include low-to-moderate doses of corticosteroids. ^( 4 , 5 )^ However, currently, the most effective and widespread measure to control the spread of the disease is vaccination, in addition to hygiene and social distancing measures. Currently, four vaccines are released and administered in Brazil. These include the following: Coronavac ^®^ , an inactivated virus vaccine, from the consortium between the Sinovac Laboratory and Butantã Institute; Covishield ^®^ , a non-replicating viral vector vaccine (NRVVV), from the consortium between the laboratory AstraZeneca and the University of Oxford/Fiocruz; Comirnaty ^®^ , an mRNA vaccine (MRNAV), produced by Pfizer Inc.; and Janssen COVID-19 ^®^ produced by Janssen Pharmaceuticals.

The literature demonstrates the safety of vaccination in patients with immunosuppression. For example, a study conducted in patients with non-Hodgkin lymphoma (NHL) treated with rituximab alone or in combination chemotherapy found 0% serum conversion at 3 weeks after vaccination to H1N1, whereas the Control Group had an 82.4% response rate. ^( 6 )^

One study compared *de novo* and recall immune responses between patients with chronic lymphocytic leukemia (CLL) who were treated with Bruton’s tyrosine kinase (IBTK) inhibitors and patients who were never treated with these inhibitors. When vaccinated with recombinant hepatitis B vaccine ( *de novo* immune response), immunization rates of 3.8% and 41.5% were observed in the treated and untreated groups, respectively. Moreover, when vaccinated with recombinant herpes zoster vaccine (recall response), rates of 41.5% and 59.1% were observed in the same groups, respectively. These results suggest that the presence of a proper humoral and cellular response prior to vaccination may contribute to a better serum conversion rate after immunization in patients administered IBTK. ^( 7 )^ In a recent study on the influence of different treatments on immunogenicity after COVID-19 vaccination, Roeker et al. determined the immune response after two doses of MRNAV in patients with CLL and found positive responses in 94% of patients who were never treated and 23% of those who had already been treated. Additionally, they observed positive responses in 21% of patients who used IBTK, 14% in those treated with anti-cluster of differentiation (CD) 20, and 0% when the monoclonal antibody was associated with Venetoclax. ^( 8 )^

Patients who underwent bone marrow transplantation had different rates of serum conversion to H1N1 vaccination according to the time after transplantation. A study conducted in patients undergoing transplantation evaluated the immune response to the H1N1 vaccine, comparing patients vaccinated before and 6 months after transplantation, and found response rates of 10-40% and 10-72%, respectively. These rates were similar to those in the healthy population 2 years after transplantation. ^( 9 , 10 )^ Serum conversion can vary according to the stage of disease progression. Patients with monoclonal gammopathy of undetermined significance (MGUS) have seroconversion rates to the pneumococcal vaccine of approximately 95%. However, only 60% of the patients with smoldering myeloma without treatment presented seroconversion after 1 month, and only 25% of them maintained the response after 12 months. In contrast, patients with multiple myeloma (MM) undergoing treatment had seroconversion rates of 43% and 14% at 1 and 6 months after vaccination, respectively. ^( 11 - 13 )^ Bird et al. analyzed the immunogenicity after two doses of COVID-19 vaccine in 93 patients with MM and found seroconversion in 56% of these patients. They also observed that the seropositivity rate varied among patients according to treatment response. They also noted that other factors, such as lymphopenia, immunoparesis, and multiple lines of treatment, were associated with low seroconversion rates. Among the patients studied, eight underwent autologous stem cell transplantation (ASCT) within 12 months of vaccination, and six had positive responses after two doses of the immunizer. ^( 14 )^

A study that measured the seroconversion to H1N1, H3N2, and influenza B in patients with CLL found rates of 58.8%, 83.8%, and 17.6%, respectively, in comparison to patients with monoclonal B-cell lymphocytosis who presented rates of 69.2%, 100%, and 76.9%, respectively. ^( 15 )^

## OBJECTIVE

To evaluate the influence of onco-hematological pathologies on seroconversion to COVID-19 vaccines, in addition to the effects of chemotherapy on this response.

## METHODS

The patients with onco-hematological diseases and Control Group were scheduled to undergo collection of two 5mL blood samples in an ethylenediamine tetraacetic acid tube before vaccination and 30 days after the second (D2nd) and third doses (D3rd) for hemogram. Total immunoglobulin (Ig) G, IgA, and IgM dosage, CD4/CD8 cell count, and IgG coronavirus serology were also assessed through chemiluminescence microparticle immunoassay for the quantitative detection of IgG antibodies against the spike protein (S) binding domain of severe acute respiratory disease coronavirus 2 (SARS-CoV-2) using Abbott’s kit SARS-CoV-2 IgG II Quant™. Comparative analysis of the data obtained according to the underlying disease, age, sex, treatment protocol performed for the oncological disease, and history of COVID-19 infection was performed.

### Statistical methods

Data analysis was performed using the SAS software version 9.4. Continuous variables were summarized using descriptive statistics, such as mean, standard deviation (SD), median, minimum, maximum, and number of valid observations. Qualitative variables were summarized using absolute and relative frequencies.

Quantitative variables were compared using nonparametric tests as follows: Mann-Whitney (comparison between two independent groups), Kruskal-Wallis (comparison among three independent groups), and Wilcoxon (comparison of repeated measures) tests. The nonparametric tests proved to be more suitable for the analysis because of the lack of proof of normality and/or homoscedasticity of the variables.

Qualitative variables between the groups were compared using χ ^2^ or Fisher’s exact test.

The significance level of the tests was set at 5%, and the conclusions in cases of multiple comparisons were controlled for using the Bonferroni method.

### Primary endpoint

The primary endpoint was the immunological response to vaccination for COVID-19 after the complete administration of the immunizer in patients with onco-hematological diseases and a Control Group, according to the vaccination schedule of the State of São Paulo.

### Secondary endpoints

The secondary endpoint was the comparison of the immune response between the studied groups according to age, sex, underlying disease, total immunoglobulin level, CD4 and CD8 cell counts, and treatment. The data obtained were compared with those found in the Control Group.

### Inclusion criteria

The inclusion criteria were as follows: patients aged >18 years, with onco-hematological diseases, and with Eastern Cooperative Oncology Group (ECOG) performance status score of 0-3 and, healthcare professionals from the *Instituto de Assistência Médica ao Servidor Público do Estado de São Paulo* (IAMSPE), aged >18 years, and without onco-hematological diseases.

All patients were followed up at the Hematology Service of the IAMSPE and signed an agreement term approved by our local ethics committee (CAAE: 45300521.8.0000.5463; # 4.997.452).

### Exclusion criteria

The exclusion criteria were as follows: patients and healthcare professionals with cardiac, pulmonary, neurological, hepatic, or severe renal comorbidities without clinical control; with ECOG performance status score >3; and who were human immunodeficiency virus-positive with a CD4 count <200.

## RESULTS

Eighty-four patients were recruited to the Hematological Group, with a median age of 65 years. This group was divided into three subgroups according to etiological diagnosis: lymphoproliferative disease (LD), MM, and myeloproliferative diseases ( Table 1 ).

**Table 1:** Demographic characteristics in subgroups studied and control

Feature	Value	

	Hematological Group	Control Group
Sex, n (%)		
Female	50 (59.5)	19 (76.0)
Male	34 (40.5)	6 (24.0)
Age, n (%)		
<60	30 (35.7)	19 (76.0)
61-70	26 (31.0)	6 (24.0)
71-80	26 (31.0)	0
>80	2 (2.4)	0
Diagnostic Hematological Group, n (%)		
Lymphoproliferative diseases and Myeloma	69 (82.14)	
NHL	35 (41.66)	
MM	17 (20.23)	
HL	6 (7.14)	
CLL	6 (7.14)	
ALL	1 (1.19)	
WM	1 (1.19)	
TL	1 (1.19)	
SP	1 (1.19)	
MGUS	1 (1.19)	
Myeloproliferative diseases	15 (17.85)	
ET	7 (8.33)	
PV	5 (5.95)	
MF	1 (1.19)	
MDS	1 (1.19)	
CML	1 (1.19)	

NHL: non-Hodgkin’s lymphoma; MM: multiple myeloma; HL: Hodgkin’s lymphoma; CLL: chronic lymphocytic leukemia; ALL: acute lymphoblastic leukemia; WM: Waldenstrom’s macroglobulinemia; TL: T lymphoma; MGUS: monoclonal gammopathy of undetermined significance; SP: solitary plasmacytoma; ET: essential thrombocythemia; PV: polycythemia vera; MF: myelofibrosis; MDS: myelodysplastic syndrome; CML: chronic myeloid leukemia.

Eight patients were excluded because of death before vaccination or withdrawal.

The Control Group consisted of healthcare professionals from the IAMSPE, totaling 25 healthy individuals with a median age of 47 years.

The Hematological Group achieved an average rate of positive vaccine response of 74.02% on the D2nd, whereas the Control Group achieved 100% response rate. When the vaccine response was classified according to age, there was a trend toward lower responsiveness at older ages.

**Figure 1:**
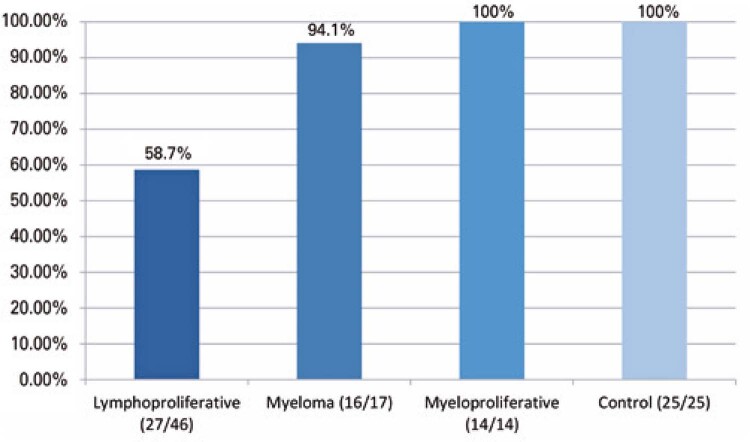
Positive vaccine response rate on the second dose in subgroups studied and Control Group (positive response/total individuals)

In the subgroup of patients with LD, an overall response rate of 58.7% was observed. In the MM group, we observed a response rate of 94.1% ( Figure 1 ).

The mean values of COVID-19 IgG, immunoglobulins, CD4, and CD8 cells according to the vaccine response in all participants are shown in table 2 . We observed that despite the large SD, the mean values of IgG COVID-19 did not differ as much in the groups studied. In the group of patients with LD, we observed a mean total IgG of 980.7 (SD±218.1) mg/dL among those with a positive vaccine response, and 738.8 (SD±393.9) mg/dL in those with a negative response (p=0.019). Interestingly, in the MM group, we observed mean total IgG levels of 1014 (SD±337.2) mg/dL in those with a positive response and a value of 7362mg/dL in the only non-responder patient.

**Table 2:** Laboratory data on the second dose according to vaccine response in subgroups studied and control

Groups	Features	
	Mean COVID-19 IgG (AU/mL)	
Lymphoproliferative Myeloma Myeloproliferative Control	3299.69 (SD±6788.63) 3459.58 (SD±3873.45) 3771.45 (SD±5854.75) 2766.3 (SD±4019.8)	
	Positive vaccinal response	Negative vaccinal response
	Mean total IgG (mg/dL), p=0.019	
Lymphoproliferative Myeloma Myeloproliferative Control	980.7 (SD±218.1) 1014 (SD±337.2) 1338.2 (SD±352.7) 1138.4 (SD±163.8)	738.8 (SD±393.9) 7362 (SD±0) 0 0
	Mean total IgM (mg/dL), p=0.459	
Lymphoproliferative Myeloma Myeloproliferative Control	65.3 (SD±45.1) 47.3 (SD±49.3) 175 (SD±188.8) 120.3 (SD±46.8)	491.8 (SD±1891.8) 14 (SD±0) 0 0
	Mean total IgA (mg/dL), p=0.142	
Lymphoproliferative Myeloma Myeloproliferative Control	191.8 (SD±94.5) 183.4 (SD±219.6) 292.3 (SD±124.8) 227.9 (SD±80.8)	170.6 (SD±145.7) 40 (SD±0) 0 0
	Mean CD4 cells/mm ^3^ , p=0.611	
Lymphoproliferative Myeloma Myeloproliferative Control	499.3 (SD±196.6) 469.2 (SD±268.9) 688 (SD±279.2) 826.9 (SD±348.4)	538 (SD±396.4) 244 (SD±0) 0 0
	Mean CD8 cells/mm ^3^ , p=0.878	
Lymphoproliferative Myeloma Myeloproliferative Control	455 (SD±208.9) 401.2 (SD±283.3) 375.1 (SD±185.3) 357.1 (SD±143)	611.1 (SD±746.5) 1297 (SD±0) 0 0

Furthermore, immunoglobulin, CD4, and CD8 cell results did not show any relationship with immune response ( Table 2 ).

We also observed in the group of patients with LD that the presence of lymphopenia was associated with an immunization rate of 85.7%, against 65.8% in those with more than 1000 lymphocytes/mm ^3^ (p=0.031).

### Vaccine response and multiple myeloma

**Figure 2:**
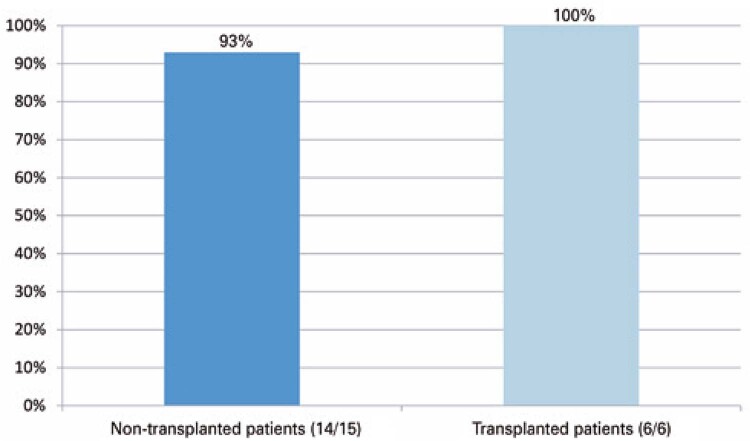
Vaccine response rate on the second dose in the myeloma subgroup, stratified in transplanted and non-transplanted patients (positive response/total individuals)

We evaluated the vaccine response in 17 patients with MM, with a mean age of 67 (SD±7.91) years, composed of 5 MM IgG kappa, two MM IgG lambda, two MM IgA kappa, one MM IgA lambda, four MM kappa light chain, and one MM lambda light chain. There were three patients with an International Staging System (ISS) of 1, 4 with ISS of 2, 7 with ISS of 3, and one without ISS, as assessed in the medical records. Regarding the treatment line, seven patients were in the first line, six in the second line, and two had already had three or more lines. We observed a positive vaccine response in 16 patients, with a mean value of IgG COVID-19 antibodies of 3459.58 (SD±3873.45) AU/mL. The only patient with a negative response received an inactivated virus vaccine. Of these 17 patients, six underwent ASCT and one underwent syngeneic stem cell transplantation. All patients had a positive vaccine response, including one patient who was vaccinated before completing 1 year of transplantation, with a mean value of IgG COVID-19 antibodies of 2695.21 (SD±2877.9) AU/mL ( Figure 2 ).

### Recall response

We analyzed the clinical and vaccination history of a group of 24 patients with LD or MM and observed that six patients reported previous contact with the virus, 15 denied it, and three were unable to respond.

Of the six patients with a history of previous infection, 4 (66.6%) had detectable COVID-19-specific IgG before vaccination evaluation before first vaccine dose (D0), with a mean value of 4302.8 (SD±5385.75) AU/mL. After vaccination, they remained positive, and their titer levels increased, reaching a mean value of 7645.1 (SD±6571.14) AU/mL. Of the two (33.3%) patients who reported previous infection, but did not have COVID-19-specific IgG on D0, only one had a positive vaccine response, with levels of 46455.6 AU/mL. The patient with NHL was in remission and was treated more than 2 years prior to vaccination with NRVVV.

In the group of 15 patients who denied previous infection, 2 (13.3%) had detectable COVID-19-specific IgG, with a mean value of 2271.65 (SD±2969.77) AU/mL in the D0, and maintained a response after the vaccination, with a mean value of 3952.1 (SD±5153.53) AU/mL. Of the 13 (86.6%) remaining patients who denied previous infection and did not have detectable COVID-19-specific IgG on D0, 8 (61.53%) had a positive vaccine response, with a mean value of 5244.71 (SD±6945.19) AU/mL.

**Figure 3:**
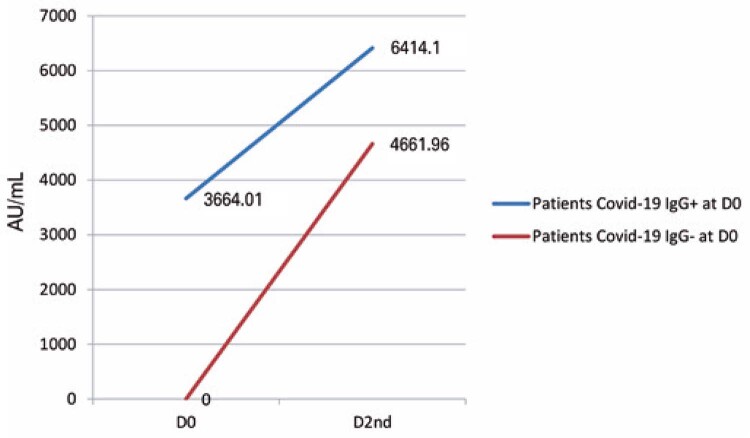
Quantitative increment in immunoglobulin (IgG) coronavirus disease (COVID-19) antibodies after vaccination in patients with lymphoproliferative diseases and multiple myeloma with detectable or not detectable IgG COVID-19 antibodies at the first vaccine dose (D0), evidencing a significant recall response

Based on these data, we found that in patients previously exposed to the virus, there was a 75% increase in their COVID-19-specific IgG values after vaccination, which was 37% higher than the average level of antibodies in patients who were not exposed to the virus before vaccination ( Figure 3 ).

We were able to evaluate the vaccine response to the booster dose in 24 patients from the Hematological Group, with 21 vaccinated with MRNAV and three with inactivated virus vaccine. These patients had mean COVID-19 IgG values of 5694.2 (SD±8025) and 32729.4 (SD±31869.7) AU/mL at the D2nd and D3rd, respectively, an approximate 5.74-fold increase (p<0.001).

### Vaccine response and anti-cluster of differentiation (CD) 20 therapy

**Figure 4:**
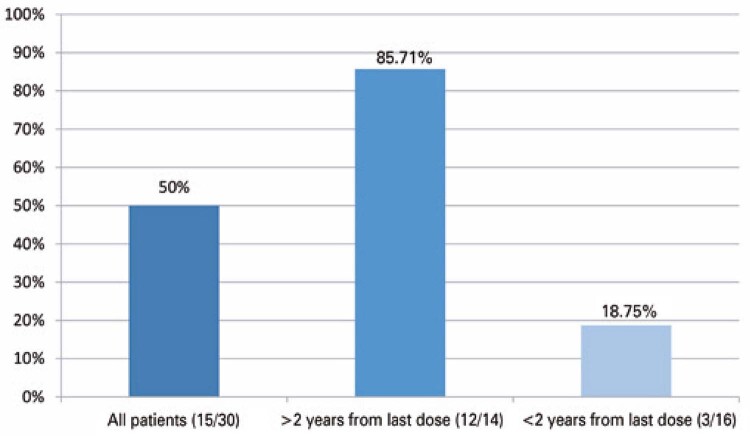
Vaccine response rate on the second dose in the lymphoproliferative disease subgroup, stratified according to time of anti-cluster of differentiation 20 therapy relative to vaccination (positive response/total individuals)

We analyzed the vaccine response of 30 patients with a history of B-cell-depleting treatment and found positive serology in 50% of them, with a mean COVID-19 specific IgG value of 9519.67 (SD±13747.04) AU/mL. The patients were also divided according to the time of anti-CD20 treatment (>2 and <2 years) and the date of vaccination. Of the 14 patients vaccinated and treated with anti-CD20 for more than 2 years before the first vaccine dose, 12 (85.71%) had a positive vaccine response on the D2nd, with a mean COVID-19-specific IgG of 11790.025 (SD±14573.30) AU/mL. Of the 16 patients vaccinated and treated for less than 2 years before the first vaccine dose, only 3 (18.75%) had a positive viral response, with a mean COVID-19-specific IgG of 438.36 (SD±374.86) AU/mL ( Figure 4 ).

## DISCUSSION

### Immunogenicity and etiological groups

Lymphoproliferative diseases tend to have lower rates of immunogenic response than other diseases. In our study, we observed an immune response rate of 58.7% in patients with LD, which was lower compared to that of patients with myeloproliferative diseases who behaved similarly to the general population. Cattaneo et al. analyzed immunogenicity after COVID-19 in a group of 45 patients with hematological malignancies and found a lower serum conversion rate after 2 months of acute infection in patients with diffuse large B-cell lymphoma and follicular lymphoma (50%) than in patients with other hematological malignancies (85%). ^( 16 )^

### Vaccine response and myeloproliferative diseases

We observed a positive vaccine response in 14 of the 15 patients with MM recruited for the study and in patients with MGUS and solitary plasmacytoma. Factors associated with lower seroconversion rates in the literature, such as lymphopenia, immunosuppression, advanced age, multiple lines of treatment, ISS >1, and vaccine platform, did not interfere with the rates in these patients.

Most studies differ regarding the vaccine responsiveness of patients with MM; however, our results tend to agree with the portion of studies that verify adequate responses in this population. For example, Avivi et al. assessed the vaccine response after MRNAV in 159 patients with MM and found positive serology in 76% of patients compared to a Control Group of 94 individuals who obtained 98% positivity. ^( 17 )^ However, Stampfer et al. found an adequate vaccine response in only 45% of 96 patients with MM who received two doses of MRNAV. ^( 18 )^

Literature data also differ regarding the vaccine response after ASCT in patients with MM.

Salvini et al. studied the vaccine response 28 days after two doses of MRNAV in 46 patients with MM who underwent ASCT at an average of 17 months before vaccination and found a 91.3% positive vaccine response in this population. ^( 19 )^ In contrast, Dhakal et al. observed the vaccine response 14 days after two doses of MRNAV or NRVVV in 30 patients with MM approximately 30 months after ASTC and found positivity in only 63% of these patients. ^( 20 )^

In our study, most patients underwent ASCT more than 6 months before vaccination, a period in which there is a greater probability of a positive vaccine response, as recommended by the European Myeloma Network. ^( 21 )^

### Recall response

Kamar et al. studied the humoral response to three doses of MRNAV in 101 patients undergoing solid organ transplantation using immunosuppressants. The prevalence rates of anti-SARS-CoV-2 antibodies after the first, second, and third doses were 4%, 40%, and 68%, respectively. ^( 22 )^ In our study, six patients who already had detectable IgG COVID-19 antibodies before vaccination achieved a positive response after the immunizer and presented a significant increase in the averages of antibodies of 3664.01-6414.1 AU/mL. In addition, we were able to demonstrate the benefit of the third booster, which provided a 5.74-fold increase in antibody levels. Our results tend to agree with the literature and indicate an important recall response in the context of COVID-19 vaccination, even in patients with immunosuppression.

### Lymphopenia

Achiron et al. studied the vaccine response 1 month after two doses of MRNAV in patients with multiple sclerosis. In the group of 26 patients treated with fingolimod, only one patient with a lymphocyte count of 700cells/mm ^3^ developed a positive humoral response. ^( 23 )^ In turn, Ducloux et al. analyzed the vaccine response 1 month after two doses of MRNAV in 50 patients undergoing dialysis without a history of COVID-19 and found antibody levels above 50 and 224 AU/mL in 90% and 74% of patients, respectively. The mean lymphocyte counts in non-responding and responding patients were 1046cells/mm ^3^ and 1363cells/mm ^3^ , respectively. ^( 24 )^

### Immunogenicity and immunosuppression

In our study, we observed an association between a higher mean total IgG level and a positive response to the vaccine in patients with LD, which is consistent with the literature. Benda et al. showed a higher serological response rate in patients with high immunoglobulin levels before vaccination, in which patients with IgG >550mg/dL had a 4.9-fold greater chance of serological response. In addition, there was a correlation between treatment and lower IgG levels, as 88.8% of patients with IgG ≤550mg/dL were undergoing chemotherapy, of whom 22 were also on targeted therapy. ^( 25 )^

### Cellular immune response

The total counts of CD4 and CD8 cells were not associated with vaccine response in our study. Tan et al. observed that patients with early proliferation of SARS-CoV-2-specific interferon gamma-secreting T-cells had milder disease and faster viral clearance. ^( 26 )^ Ehmsen et al. observed a worse cellular immune response in patients with onco-hematological diseases 36 days after the administration of two doses of MRNAV. In the population studied, only 215 (66%) patients produced anti-spike IgG antibodies. In this same population, when performing immunoassays for interferon gamma release, a positive T-cell response was observed in only 45% of the patients, and 81% of these were positive for both CD4+ and CD8+ cells. When correlating humoral and cellular responses, 74% of seronegative patients had no T response. ^( 27 )^

### Vaccinal response and anti-CD20 therapy

The time between the use of anti-CD20 monoclonal antibodies and vaccination seems to affect the humoral immune response. In our study, only 18.75% of patients who received anti-CD20 therapy for <2 years before the vaccination schedule had a positive vaccine response, compared to 85.71% of patients who completed treatment for >2 years before vaccination, which is in agreement with the literature. Ollila et al. verified the vaccine response to COVID-19 in 105 patients who received B-cell-depleting monoclonal antibodies and found a 29% positive vaccine response rate. Among patients who completed chemotherapy >12 months prior to vaccination, 69% were seroconverted, compared to 24% among those who were vaccinated within 12 months of the last treatment. ^( 28 )^ Deepak et al. observed that the lack of seroconversion after anti-CD20 treatment occurred mainly in those who received vaccination within 6 months of administration, with gradual recovery of the antibody response to vaccination 9 months after treatment with rituximab. ^( 29 )^

In our study, we had two patients using ibrutinib with more than four lines of treatment: one with Waldenstrom’s macroglobulinemia and another with CLL. Both patients received inactivated virus vaccine, and only the patient with CLL showed a positive response after 30 days of vaccination. Parry et al. studied the vaccine response to MRNAVs and NRVVV in patients with CLL and found only 21% of positive responses in the subpopulation treated with IBTK after 5 weeks of the immunizing agent first dose. However, this study has not yet been completed. ^( 30 )^

## CONCLUSION

Compared with other patients, those with lymphoproliferative disorders are the most vulnerable and likely to progress to more severe cases of COVID-19 and to have lower seroconversion rates, both due to the biology of the disease and lymphodepleting treatment. Several studies have observed lower humoral responses in this population, especially in patients with diffuse large B-cell lymphoma, follicular lymphoma, and chronic lymphocytic leukemia.

The group of patients with multiple myeloma deserves to be highlighted since the literature shows divergence regarding its immunogenicity. Our results showed adequate responses in this population, and factors traditionally associated with lower seroconversion rates did not interfere.

The most interesting result of our study is the immunogenic recall response in patients with lymphoproliferative disease. We observed that patients previously exposed to COVID-19 had higher antibody values 30 days after vaccination than their pre-vaccination values, and that these same values were higher than those of patients who did not have such exposure. In addition, we demonstrated a significant increase in antibody levels after the third dose of immunization, reinforcing the benefits of the booster dose.

Lymphodepletive therapy has also been shown to be a determinant of the immune response in patients with lymphoproliferative disorders. We found that patients who had received treatment for >2 years before the vaccination schedule had higher rates of positivity than those in treatment for <2 years.

Initially, our study aimed to observe the vaccine responses of patients with onco-hematological diseases at 30 and 180 days, thus evaluating the initial antibody response and the senescence behavior. However, the protocol needed to be adapted when the third booster dose was included in the vaccination schedule of São Paulo, which made the long-term evaluation of antibodies unfeasible, which would be particularly important in the context of the emergence of new virus variants.

The low sampling rate made it difficult to assess the immunogenicity of patient subgroups, showing the need to expand these groups and follow-up. However, we were able to verify that patients with onco-hematological diseases benefit from vaccination and vaccine booster, regardless of the platform used. We also examined the safety of these platforms, as we did not record any severe vaccine reactions.
